# Ensuring the Nation’s Mental Health: The Role of Federal Agencies

**Published:** 2009-12-15

**Authors:** A. Kathryn Power

**Affiliations:** Substance Abuse and Mental Health Services Administration

Behavioral health care, which addresses mental health and substance use, has been neglected by our health care system. People with severe mental illnesses and other chronic conditions such as diabetes, asthma, heart disease, and obesity, die earlier than do people without such conditions. Behavioral health conditions may not be properly addressed because of a lack of community resources and poor health insurance coverage for such conditions. The federal government — in partnership with states, communities, consumers, families, and the private sector — has responded with proposals for health care reform that include mental health care.

In 2009, federal agency partners helped frame the agenda for health care reform. Their focus was on creating a holistic, person-centered health care system, achieved through a public health model that addresses the broad determinants of health and mental health. In particular, they focused on prevention, early intervention, and the use of technology to increase access to and quality of health and mental health services.

The federal response to behavioral health care reform has been guided by 3 seminal documents that have influenced and reflected change in mental health systems and services. Reports by the Surgeon General (1), New Freedom Commission on Mental Health (2), and Institute of Medicine (3) made all of the following clear:

- Mental health is essential to overall health.
- Mental illnesses are real disorders and treatment is effective.
- Recovery is the expected outcome of treatment and services.
- Services must be consumer- and family-driven and evidence-based.

Soon after the release of the New Freedom Commission's final report in 2003, the US Department of Health and Human Services (HHS) charged its Substance Abuse and Mental Health Services Administration (SAMHSA) with leading efforts to transform the mental health system in this country. SAMHSA organized a collaborative effort among more than 20 federal agencies and offices to ensure that people with mental and substance use disorders have every opportunity for recovery. Along with other agencies and offices in HHS, the federal partners include the US departments of agriculture, defense, education, housing and urban development, justice, labor, transportation, and veterans affairs, as well as the Social Security Administration and the Equal Employment Opportunity Commission. The federal partners are guided in their work by the Federal Executive Steering Committee, a group of assistant secretary-level professionals who ensure that resources will be available and promised actions will occur.

Efforts to improve systems of care for people with mental illnesses have been extensive. Together, SAMHSA and its federal partners developed a specific agenda to create consumer-centered, recovery-focused, evidence-based, quality-driven systems of care for people with mental health problems. *Transforming Mental Health Care in America: The Federal Action Agenda:* *First Steps* (4), released in 2005, and *Transforming Mental Health Care in America: The Federal Action Agenda: "A Living Agenda"* (5), published in 2008, chart a vision for a transformed mental health system, delineate specific objectives to achieve this vision, and highlight progress made.

The Federal Executive Steering Committee has focused on critical issues to ensure both full community integration for people with mental health problems and broad recognition of the role of mental health in overall health. Some of these issues are countering the social exclusion of people with mental illnesses through public education, preventing suicide, and increasing employment opportunities for people with psychiatric disabilities, mental illnesses, and emotional disturbances. Efforts to expand the reach of care include collaborating with the criminal and juvenile justice systems to provide access to mental health care, addressing the mental health needs of marginalized groups such as refugees and homeless people, and improving mental health care services for children and adolescents.

Research is needed to understand concurrent mental and physical disorders, develop new medications, promote evidence-based mental health best practices, and put research findings into practice. The committee's accomplishments include a collection of federal resources on disaster behavioral health, a compendium of primary care and mental health integration initiatives across participating federal agencies, and a research study on evidence-based programs that integrate primary care and mental health.

Meeting the needs of people with existing mental illnesses is a challenge. Countering discrimination, undoing the fragmentation of services for physical and mental illnesses, and managing costs and limited resources are first steps toward a transformed mental health system. Wisely investing in the future while making the best use of limited resources will enable us to share the hope of recovery.
